# Supplementary material for: DNA Methylation at ATP11A cg11702988 Is a Biomarker of Lung Disease Severity in Cystic Fibrosis: A Longitudinal Study
Source: Genes (Basel). 2021 Mar 19;12(3):441. doi: 10.3390/genes12030441 (PMC8003783; doi:10.3390/genes12030441)
Supplement: Supplementary file 1 [file genes-12-00441-s001.zip › Supplementary/Table S1.docx]

**Table S1. Pyrosequencing primers.**

| CpG, Gene | Primers 5' → 3' (B = biotinylated) | Annealing temperature |
| --- | --- | --- |
| cg05524038 *CSF1R* | Forward: TTTTGTGGAGTTAGAAAAGTTTG  Reverse: AATAAAACACATAAAACTCACCC (B)  Sequencing: GGTTTAGGTTTTTTAGTTTTTA | 60 |
| cg06048354 *PCDHβ4* | Forward: TGGTTAAGGATTTGGGTTTGGGAA (B)  Reverse: ATTTCTCCCTCAAAAACAAATCTCC  Sequencing = Reverse | 60 |
| cg08379987 *C13orf26* | Forward: GAGTATGTTTAATTGAGGAAAAG (B)  Reverse: TATCCCCTCTTAACCAAACAC  Sequencing = Reverse | 60 |
| cg10582608 - | Forward: GTGTTTTTTAGTTTAGAGGTAG  Reverse: CAATCTCAAAACCTAAAACTC (B)  Sequencing: GATTTTTATATTATGTTGG | 56 |
| cg11702988 *ATP11A* | Forward: GTGGTGGTGGGTTTGATT  Reverse: AACCACACCTAAATACTAC (B)  Sequencing: GAGGTTTATTTTTGTTATTTG | 54 |
| cg17735593 *PCDHβ7* | Forward: GTGTTGTGTAGAAAAGGTAAG  Reverse: CCTACCCCTAACCCTAAATCT (B)  Sequencing: GGGAATGTTTTGGGTTGG | 58 |
| cg23299919 *PTPRN2* | Forward: TGGGTTTGAGGTGATATGTTTTG (B)  Reverse: CAACTCAACAAATAACTTATTCCC  Sequencing: AACACAAAACCAACAATTCC | 60 |
