# Supplementary material for: DNA Methylation at ATP11A cg11702988 Is a Biomarker of Lung Disease Severity in Cystic Fibrosis: A Longitudinal Study
Source: Genes (Basel). 2021 Mar 19;12(3):441. doi: 10.3390/genes12030441 (PMC8003783; doi:10.3390/genes12030441)
Supplement: Supplementary file 1 [file genes-12-00441-s001.zip › Supplementary/Figure S1.pdf]

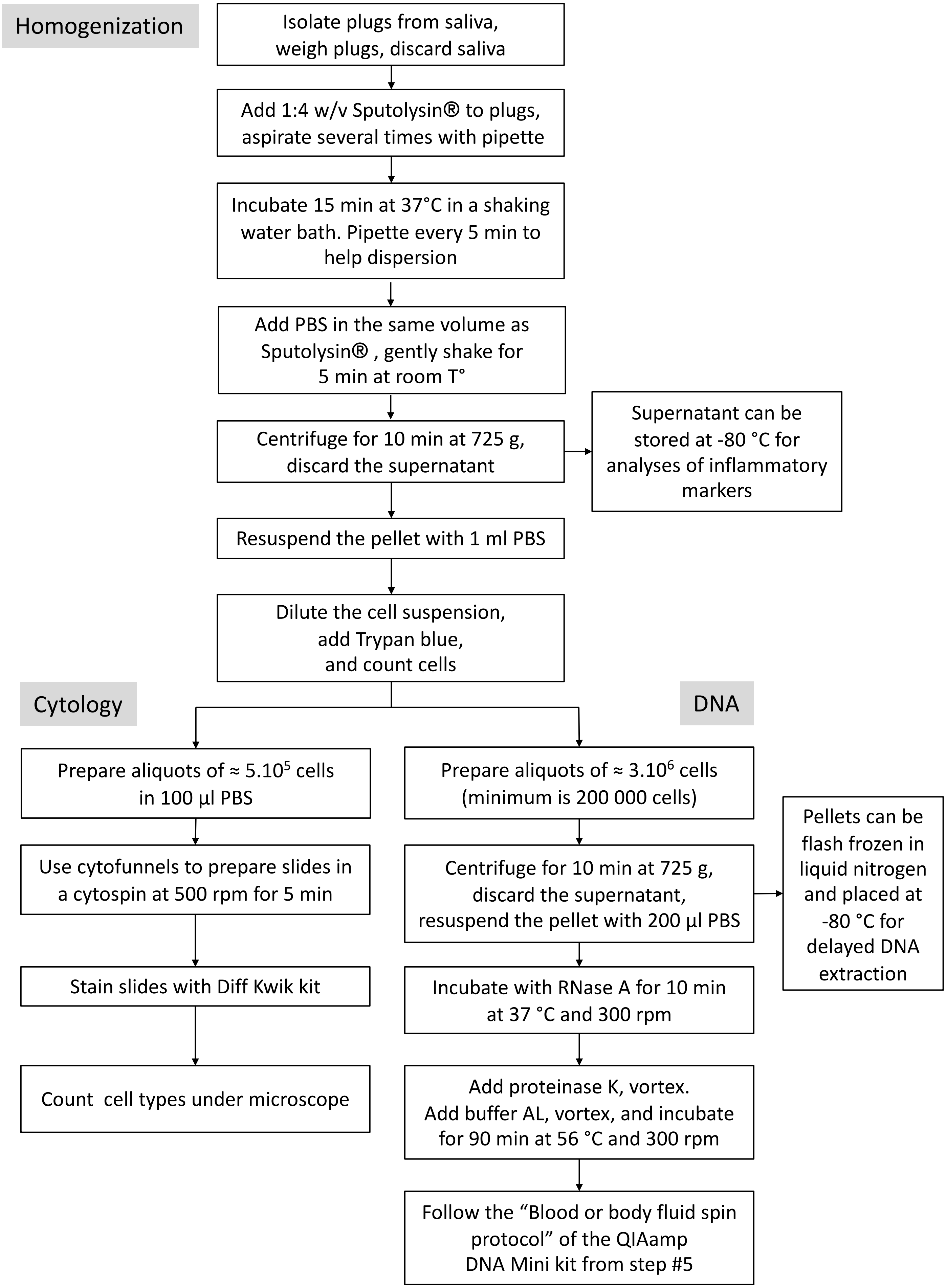
Homogenization
Isolate plugs from saliva, weigh plugs, discard saliva
Add 1:4 w/v Sputolysin® to plugs, aspirate several times with pipette
Incubate 15 min at 37°C in a shaking water bath. Pipette every 5 min to help dispersion
Add PBS in the same volume as Sputolysin® , gently shake for 5 min at room T°
Centrifuge for 10 min at 725 g, discard the supernatant
Supernatant can be stored at -80 °C for analyses of inflammatory markers
Resuspend the pellet with 1 ml PBS
Dilute the cell suspension, add Trypan blue, and count cells
Cytology
Prepare aliquots of ≈ 5.10^5 cells in 100 µl PBS
Use cytofunnels to prepare slides in a cytospin at 500 rpm for 5 min
Stain slides with Diff Kwik kit
Count cell types under microscope
DNA
Prepare aliquots of ≈ 3.10^6 cells (minimum is 200 000 cells)
Centrifuge for 10 min at 725 g, discard the supernatant, resuspend the pellet with 200 µl PBS
Pellets can be flash frozen in liquid nitrogen and placed at -80 °C for delayed DNA extraction
Incubate with RNase A for 10 min at 37 °C and 300 rpm
Add proteinase K, vortex. Add buffer AL, vortex, and incubate for 90 min at 56 °C and 300 rpm
Follow the “Blood or body fluid spin protocol” of the QIAamp DNA Mini kit from step #5
